# Comparative policy analysis of national rare disease funding policies in Australia, Singapore, South Korea, the United Kingdom and the United States: a scoping review

**DOI:** 10.1186/s13561-024-00519-1

**Published:** 2024-06-19

**Authors:** Qin Xiang Ng, Clarence Ong, Kai En Chan, Timothy Sheng Khai Ong, Isabelle Jia Xuan Lim, Ansel Shao Pin Tang, Hwei Wuen Chan, Gerald Choon Huat Koh

**Affiliations:** 1 Saw Swee Hock School of Public Health, National University of Singapore and National University Health System, Singapore, Singapore; 2 NUS Yong Loo Lin School of Medicine, National University, Singapore, Singapore; 3 Department of Ophthalmology, National University Hospital, Singapore, Singapore

**Keywords:** Rare diseases, Orphan drugs, Funding, Health policy, Risk-sharing

## Abstract

**Background:**

Rare diseases pose immense challenges for healthcare systems due to their low prevalence, associated disabilities, and attendant treatment costs. Advancements in gene therapy, such as treatments for Spinal Muscular Atrophy (SMA), have introduced novel therapeutic options, but the high costs, exemplified by Zolgensma® at US$2.1 million, present significant financial barriers. This scoping review aimed to compare the funding approaches for rare disease treatments across high-performing health systems in Australia, Singapore, South Korea, the United Kingdom (UK), and the United States (US), aiming to identify best practices and areas for future research.

**Methods:**

In accordance with the PRISMA-ScR guidelines and the methodological framework by Arksey and O’Malley and ensuing recommendations, a comprehensive search of electronic databases (Medline, EMBASE, and Cochrane) and grey literature from health department websites and leading national organizations dedicated to rare diseases in these countries was conducted. Countries selected for comparison were high-income countries with advanced economies and high-performing health systems: Australia, Singapore, South Korea, the UK, and the US. The inclusion criteria focused on studies detailing drug approval processes, reimbursement decisions and funding mechanisms, and published from 2010 to 2024.

**Results:**

Based on a thorough review of 18 published papers and grey literature, various strategies are employed by countries to balance budgetary constraints and access to rare disease treatments. Australia utilizes the Life Saving Drugs Program and risk-sharing agreements. Singapore depends on the Rare Disease Fund, which matches public donations. South Korea’s National Health Insurance Service covers specific orphan drugs through risk-sharing agreements. The UK relies on the National Institute for Health and Care Excellence (NICE) to evaluate treatments for cost-effectiveness, supported by the Innovative Medicines Fund. In the US, a combination of federal and state programs, private insurance and non-profit support is used.

**Conclusion:**

Outcome-based risk-sharing agreements present a practical solution for managing the financial strain of costly treatments. These agreements tie payment to actual treatment efficacy, thereby distributing financial risk and promoting ongoing data collection. Countries should consider adopting and expanding these agreements to balance immediate expenses with long-term benefits, ultimately ensuring equitable access to crucial treatments for patients afflicted by rare diseases.

**Supplementary Information:**

The online version contains supplementary material available at 10.1186/s13561-024-00519-1.

## Introduction

A rare disease is characterized by its low prevalence within the population. In the United States (US), a disease is classified as rare if it impacts fewer than 200,000 individuals [1]. In contrast, Japan sets this threshold at 50,000 individuals, while Australia defines a rare disease as one affecting fewer than 2,000 individuals. These criteria generally correspond to prevalences ranging from 1 to 8 per 10,000 people [2]. Singapore’s definition specifies a rare disease as one affecting less than one in 2,000 patients [3]. A significant number of rare diseases lead to fatal outcomes, and the majority have their roots in genetics, stemming from mutations in genes or chromosomes [4].

Previously, many of these rare diseases had only symptom-relieving treatments [5]; today, with the advent of gene therapy, large strides have been made with novel treatments that significantly improve one’s quality of life [6]. Using the example of Spinal Muscular Atrophy (SMA), which if left untreated, progressively weakens muscles and can lead to severe physical disabilities and death [7]. For a long time, Risdiplam stood as the treatment of choice for SMA. For those undergoing this treatment, it necessitates a daily oral regimen that extends throughout their lifetime [8]. In Singapore, the average annual cost of Risdiplam in public healthcare institutions is approximately S$375,000 [8]. There are no further subsidies by the Singaporean government and SMA treatment is not currently covered by the Rare Disease Fund (RDF). Since April 2023, Zolgensma® has been approved by local Health Sciences Authority (HSA) under the Register of Class 2 Cell, Tissue or Gene Therapy Products for use in Singapore [9]. Zolgensma®, however, costs around US$2.1 million per dose [10], and the staggering cost poses an immense financial barrier for the vast majority of patients and their families [11]. In the US and the United Kingdom (UK)/European Union (EU), Spinraza® (nusinersen) was the first approved drug for SMA [12], and its approval set a precedent for subsequent SMA treatments, including Zolgensma®, which was mostly recommended for reimbursement as an alternative.

The rarity and high cost of these treatments pose unique challenges for healthcare policy and funding. Policymakers must balance the ethical imperative to provide access to life-saving treatments with the practical constraints of healthcare budgets. Different countries have adopted various strategies to manage these challenges. For example, Australia utilizes the Life Saving Drugs Program (LSDP) and risk-sharing agreements [13], while Singapore relies on a rare disease fund that matches public donations [2]. South Korea’s National Health Insurance Service (NHIS) covers certain orphan drugs based on cost-effectiveness analyses [14], the UK employs the National Institute for Health and Care Excellence (NICE) to evaluate treatments for cost-effectiveness [15], and the US combines federal and state programs with private insurance and non-profit support [16].

This scoping review thus aimed to compare and contrast the approaches to funding rare disease treatments across high-performing health systems in Australia, Singapore, South Korea, the UK, and the US. By examining the existing policies, approval processes, and reimbursement mechanisms in these countries, this review seeks to identify best practices, learning points and potential areas for improvement in ensuring equitable access to treatment for patients with rare diseases. In addressing rare diseases, it is crucial to establish a suitable financing structure to avoid creating a schism between families who can afford these treatments and those who cannot. This also raises a parallel question on whether the price of rare disease therapies is justified, and how health systems can provide equitable treatment access for all patients, regardless of the rarity of their condition and the cost of treatment.

## Methods

### Country selection

To ensure a comparable assessment, countries with similar high-income status (based on the World Bank Group country classification) were chosen [17]. The countries selected for comparison were: Australia, Singapore, South Korea, the UK and the US. These are all countries with advanced economies, high-income economy with a high GDP per capita [17], and with high-performing health systems. Low- and middle-income countries (LMICs) were not sampled as formal health technology assessment is typically lacking or limited in these settings [18].

### Search strategy

This scoping review protocol adhered to the PRISMA-ScR (Preferred Reporting Items for Systematic Reviews and Meta-Analyses extension for Scoping Reviews) guidelines [19] and the methodological framework outlined by Arksey and O’Malley [20], as well as further recommendations made by Levac et al. [21]. To this end, a comprehensive search, encompassing both electronic databases and internet-based sources, was performed independently by five authors (CO, KEC, TSKO, IJXL and ASPT), with any discrepancies resolved by the senior author (QXN).

### Electronic database search

Using combinations of relevant key words including ‘orphan disease’, ‘rare disease’ and ‘orphan drugs’, we searched Medline, EMBASE, and Cochrane databases for studies published up to 31 May 2024. The full search strategy is displayed in the supplementary (Table S1). The search focused on published original research articles, reviews, policy papers, and government reports related to rare disease funding and policy. Inclusion criteria encompassed studies that detailed drug approval processes, reimbursement decisions and funding mechanisms in the five high-income countries (Australia, Singapore, South Korea, the UK and US), and published in the last decade (published during or after 2010) to ensure it covers recent policy changes. Exclusion criteria included non-English studies and editorial/opinion pieces lacking substantial data.

### Internet-based search

In addition to the database search, grey literature was searched via the health department websites of these five countries (Australia, Singapore, South Korea, the UK, and US) to source government reports and monographs related to rare disease policy and funding. Following this, the websites of leading national organizations dedicated to rare diseases in each country were also screened to gather more reports. The main internet sources used for data collection are listed in Table 1. The search terms employed included ‘rare diseases’ or ‘orphan diseases’ along with related phrases (such as ‘specialized care’, ‘health policy’, ‘patient advocacy’, ‘treatment access’, ‘healthcare quality’, and ‘government support’), linked by the conjunction ‘or’. The review encompassed reports published up to 31 January 2024, limited to documents published in English or translatable to English using web Google translate. The translation pertained to documents in Korean, and the Google translation was cross-checked with ChatGPT and also manually verified by a native speaker of the language.

**Table 1:** Internet sources for identification of rare disease funding-related reports in countries reviewed

Country/Organisation	Internet address
**Australia**	
Department of Health and Ageing	http://www.health.gov.au/
Medicare Benefits Schedule	https://www.mbsonline.gov.au/
Rare Awareness Rare Education (RARE)	https://rareportal.org.au/
Rare Voices Australia	https://rarevoices.org.au/
Therapeutic Goods Administration	https://www.tga.gov.au/
**Singapore**	
Agency for Care Effectiveness (ACE)	https://www.ace-hta.gov.sg/
Health Sciences Authority (HSA)	https://www.hsa.gov.sg/
Ministry of Health Singapore	https://www.moh.gov.sg/
Rare Disease Fund	https://www.kkh.com.sg/giving/Documents/Rare-Disease-Fund/index.html
Rare Diseases Society (Singapore)	https://www.rdss.org.sg/
**South Korea**	
Korea Disease Control and Prevention Agency	https://www.kdca.go.kr/
Ministry of Health and Welfare	https://www.mohw.go.kr/eng/index.jsp
Rare Genomics Korea	https://www.raregenomics.org/korea
SNUH Rare Disease Center	https://raredisease.snuh.org/
**UK**	
Beacon	https://www.rarebeacon.org/
Department of Health and Social Care	https://www.gov.uk/government/organisations/department-of-health-and-social-care
Genetic Alliance UK	https://geneticalliance.org.uk/
National Institute of Health and Care Excellence, UK	https://www.nice.org.uk/
Rare Disease UK	https://www.raredisease.org.uk/
Scottish Medicines Consortium	https://www.scottishmedicines.org.uk/
**US**	
Centers for Medicare and Medicaid Services	https://www.cms.gov/
Food and Drug Administration	https://www.fda.gov/
Institute for Clinical and Economic Review	https://icer.org/
National Conference of State Legislatures	https://www.ncsl.org/
National Institutes of Health	https://www.nih.gov/
National Organization for Rare Diseases (NORD)	https://rarediseases.org/

### Scope of review

Given the intricacies within the rare disease policy ecosystem, the analysis was confined to the processes of listing and reimbursing orphan drugs, as well as any special funding mechanisms available for such drugs. Hence, government initiatives focusing on advancing research and development (R&D) for new orphan drugs, elevating awareness and diagnostic capabilities for rare diseases, and the influence of rare disease advocacy groups’ social and political capital were not within the scope of this discussion.

### Data analysis and synthesis

The narrative synthesis approach was chosen as it allowed for a flexible yet rigorous analysis of the diverse study types and reporting, accommodating the broad range of research and policy documents included in our scoping review. Briefly, the data analysis and synthesis process were rooted in best practices for narrative synthesis, as outlined by Popay et al. [22], as we attempted to integrate findings from individual studies to produce a cohesive interpretation. This process began with a preliminary synthesis, forming an initial understanding of the data. We then explored relationships within and between reports to identify patterns, trends, and differences. This exploration included grouping studies by methodology, outcomes, and specific aspects of rare disease funding policies.

## Results

**Fig. 1:**
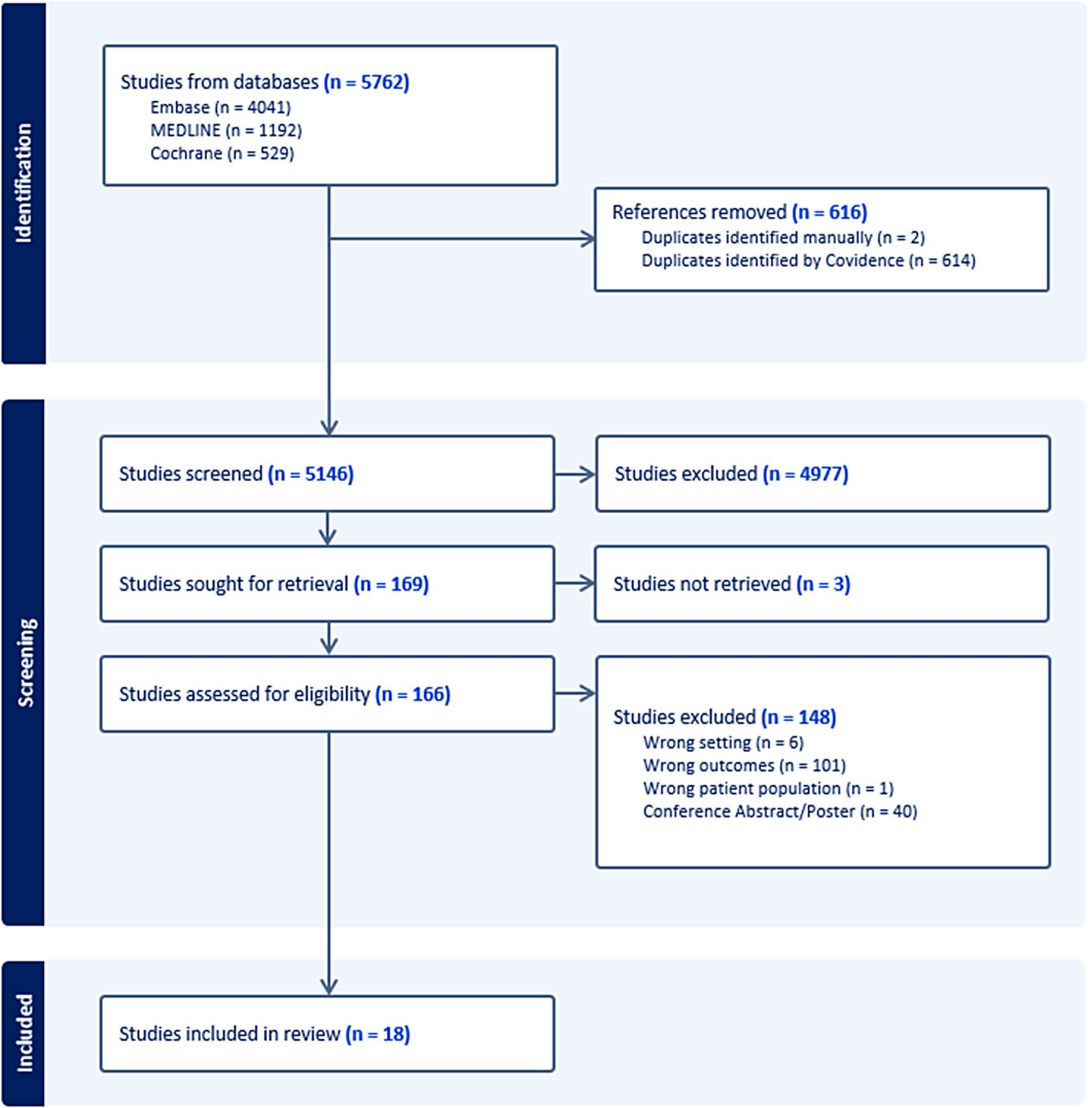
PRISMA flowchart showing the literature search process

From an initial search of Medline, Embase and Cochrane database, 5762 articles were found. After removal of 616 duplicates, 5146 articles were assessed in the title and abstract sieve. A total of 169 studies were eventually sought for full-text screening, resulting in a final selection of 18 articles for this study [23–40]. All studies were published from 2011 to 2023. The search and abstraction process are illustrated in Fig. 1, and the key study findings are summarised in Table 2.

**Table 2:** Summary of key findings from studies reviewed

Country	Study	Condition(s)	Funding policies	Approval processes	Challenges/ Barriers	Outcomes	Innovations/ Recommendations
**Australia**	Blankart et al. [23]	CML, HAE, Fabry’s disease	Co-payment, reimbursement	Annual co-payment thresholds, concession cards	High out-of-pocket costs	Subsidized treatments for specific conditions	Simplified reimbursement procedures
	Chan et al. [24]	General	Orphan drug fee waivers	Waiver of application and registration fees	High application costs	Reduced financial burden for manufacturers	Encouragement of orphan drug development
	Degtiar et al. [25]	General	Managed entry agreements, national registries	Company-funded validation of outcomes	Validation of uncertain outcomes	Adjusted pricing based on real-world performance	MEAs for managing risk
	Gammie et al. [26]	General	Financial incentives, LSDP	Fee reductions for marketing authorization	High costs of orphan drugs	Improved access through financial incentives	Implementation of outcome-based agreements
	Huang et al. [27]	General	Pharmaceutical Benefits Scheme, LSDP	Evaluated by TGA, ARTG, and PBAC	Cost-effectiveness concerns for orphan drugs	Subsidized access to orphan drugs	Enhanced access through government programs
**Singapore**	Degtiar et al. [25]	General	Rare Disease Fund (RDF)	Community and government matching contributions	High reliance on public donations	Financial support for rare disease patients	Expansion of RDF to cover more conditions
	Gammie et al. [26]	General	Free market pricing, reimbursement procedures	Simplified evaluation by the Centre for Drug Administration	Lack of orphan drug legislation	Increased drug accessibility	Streamlining drug evaluation processes
**South Korea**	Bang et al. [28]	Specific Rare Diseases	NHIS, ED pathway, PE pathway, RSA	Reimbursable price evaluation, consensus on budget impact	Stringent criteria for high-priced therapies	Lower copayment rates for rare diseases	Policy changes for high-priced therapies
	Degtiar et al. [25]	General	Legislation similar to US Rare Disease Act	National Fundholding System for Rare Disease	Legislative similarities	Structured support system for rare disease treatments	Development of rare disease-specific funding policies
	Lee et al. [29]	General	HTA, orphan drug regulations	Managed pharmaceutical expenditure, flexible ICER thresholds	High pharmaceutical expenditure	Improved access through legislative benefits	Reimbursement policies considering orphan drug characteristics
	Lee et al. [30]	Multiple conditions	Government incentives, premium pricing	Pricing based on A7 countries, RSA for premium pricing	High R&D and trial costs	Significant price reductions over time	Flexible P&R policies to manage budget impact
	Song et al. [31]	General	Orphan Drugs Guideline (2003)	Medical expense reimbursement	Limited access to essential drugs	Improved access through government support	Expansion of orphan drug guidelines
**UK**	Abbas et al. [32]	General	EU Orphan Drug Legislation	Market exclusivity, financial incentives, tax exemptions	Accessibility issues	Improved orphan drug access	Adoption of value-based pricing system
	Blankart et al. [23]	CML, HAE, Fabry’s disease	Managed by NHS, regional decisions	Variable local approval processes	Improved funding for cost-effective treatments	Streamlined access to essential drugs	NS
	Gammie et al. [26]	General	Price regulation, reimbursement procedures	PPRS rate of return limits, ICER criteria	Regulatory and financial constraints	Enhanced funding for cost-effective treatments	Implementation of value-based pricing for new medicines
	Mikami et al. [33]	Multiple conditions	Patient support programs, transparency	High treatment costs	Increased access through patient support programs	Coordination of post-market HTA and access processes	NS
	Picavet et al. [34]	General	HTA, managed entry agreements	Cost-effectiveness criteria	High treatment costs	No direct association between treatment costs and pricing system	Coordination of post-market HTA and access processes
	Stawowczyk et al. [35]	General	Highly Specialised Technologies (HST) program	Automatic funding for ultra-rare conditions	High cost-effectiveness thresholds	Significant reimbursement for rare conditions	Expansion of HST program
	Song et al. [31]	General	Price Regulation	Profit control to constrain prices	NS	NS	NS
**US**	Abbas et al. [32]	General	Orphan Drug Act	Market exclusivity, financial incentives, tax exemptions	Limited accessibility for high-cost therapies	Improved access and funding for orphan drugs	Adoption of outcomes-based agreements for high-cost therapies
	Annemans et al. [36]	NMDs	Private and public insurance	Coverage based on clinical requirements	Coverage restrictions	Variable access depending on insurance plans	Outcomes-based agreements for expensive therapies
	Blankart et al. [23]	CML, HAE, Fabry’s disease	Medicare-approved health plans	High co-pays for patients	Limited access due to high out-of-pocket costs	Reduced financial burden through Medicare coverage	Expansion of patient assistance programs
	Degtiar et al. [25]	General	Medicare 20% co-insurance rate	High patient co-pays	Limited access to orphan drugs due to high co-pays	Increased spending on orphan drugs	Continued growth in orphan drug expenditure
	Doshi et al. [37]	MS, CML	Medicare Part D	High coinsurance for specialty drugs	High out-of-pocket costs	Decreased treatment interruptions	Introduction of annual out-of-pocket spending limits
	Garland et al. [38]	MIS, RCTs	Affordable Care Act	No significant increase in MMS utilization	Inconsistent coverage across plans	Improved diagnosis and treatment outcomes	Continued evidence generation for coverage policies
	Gammie et al. [26]	General	Financial incentives, reimbursement procedures	Tax credits, Medicare fee waivers	High costs and co-pays	Improved access through financial incentives	Enhanced Medicare reimbursement processes
	Huang et al. [27]	General	ACA, Medicare Part D	Evidence-based Medicare decisions	High patient co-pays	Improved access through Medicare	Evidence-based reimbursement policies
	Lima et al. [39]	ICC	Affordable Care Act	Medicare reimbursement, expanded Medicaid eligibility	High costs and co-pays	Improved early-stage diagnosis and treatment outcomes	Continuous evidence generation for coverage policies
	Margaretos et al. [40]	SMA, ALS, DMD	Private insurance	Coverage restrictions based on clinical requirements	Variable access across insurance plans	Inconsistent coverage policies	Research on public vs. private coverage for NMD DMTs
	Mikami et al. [33]	Multiple conditions	Patient support programs, transparency	High treatment costs	Increased access through patient support programs	Coordination of post-market HTA and access processes	NS
	Song et al. [31]	General	Pricing, Reimbursement Procedures, Orphan Drug Act (1983), Rare Diseases Act (2002)	Free market pricing, Medicaid, Veterans Health Administration and Pharmacy reimbursement	NS	NS	NS

*Abbreviations* $A, Australian Dollars; $US, United States Dollars; ACA, Affordable Care Act; AJCC, American Joint Committee on Cancer; ATRG, Australian Register of Therapeutic Goods; CML, Chronic Myeloid Leukemia; ED, Essential Drug; EMA, European Medicines Agency; EU, European Union; FDA, Food and Drug Administration; HTA, Health Technology Assessment; ICC, Intrahepatic Cholangiocarcinoma; ICER, Incremental Cost-Effectiveness Ratio; LSDP, Life Saving Drugs Program; MIS, Melanoma-In-Situ; MMS, Moh’s Micrographic Surgery; NHS, National Healthcare System; NICE, The National Institute for Health and Care Excellence; NS, not specified; PBAC, Pharmaceutical Benefits Advisory Committee; PBS, Pharmaceutical Benefits Scheme; PE, Pharmacoeconomics Exemption; PPRS, Pharmaceutical Price Regulation Scheme; QALY, Quality Adjusted Life Year; QL, Quantity Limits; R&D, Research and Development; RCT, Rare Cutaneous Tumours; RDF, Rare Disease fund; RSA, Risk Sharing Agreement; SP, Special Program; TGA, Therapeutic Goods Administration; WAP, Weighted Average Price

After reviewing the internet-based sources, the salient features and comparison for the countries reviewed are holistically considered and summarised in Table 3.

**Table 3:** A comparison of rare disease funding approaches of Australia, Singapore, South Korea, the UK and US

Aspect/Country	Australia	Singapore	South Korea	UK	US
**Healthcare System**	Regionally administered, universal public health insurance program (Medicare)	Mixed financing system (premiums, deductibles, co-insurance and co-payment)	National Health Insurance Service	Publicly funded (NHS)	Mixed public and private
**Funding Model for Rare Diseases**	Medicare, PBS, LSDP	RDF, public donations, government matching	NHIS, positive listing approach	NHS, HST Programme, IMF	Private insurance (most American children are covered by their parents’ health plan), Medicaid/Medicare
**Treatment Coverage**	Subsidizes high-cost drugs for > 10 rare diseases	Limited to very specific conditions and medications	Covers certain orphan drugs based on cost-effectiveness analyses	Evaluates treatments for cost-effectiveness, funds approved and NICE-recommended treatments	Various programs and insurance coverages, large number of ongoing clinical trials
**Drug Approval and Reimbursement Criteria**	TGA approves drugs; PBAC evaluates for reimbursement based on clinical effectiveness, cost-effectiveness, and potential impact on the healthcare system	HSA approves drugs; ACE evaluates for subsidy based on clinical and economic evidence, ethical and social considerations	MFDS approves drugs; HIRA and NHIS manage reimbursement with risk-sharing agreements for high-cost drugs; pharmacoeconomic evaluation may be exempt for certain drugs	MHRA approves drugs; NICE evaluates for cost-effectiveness and clinical benefit, uses HST criteria for rare diseases	FDA approves drugs and can grant drugs orphan drug designation; pricing and reimbursement influenced by negotiations among pharmacy benefit managers, insurers, and healthcare providers
**Out-of-Pocket Expenses**	Low	High	Low	Low	Varied, often high and dependent on employment insurance
**Challenges**	Balancing budget and access to rare disease drugs	High reliance on public donations, limited RDF scope and coverage	Stringent criteria for rare disease drug reimbursement	Complicated health technology assessment process; less than 50% of centrally authorised rare disease treatments are routinely funded	Complex insurance system, high out-of-pocket costs
**Sustainability**	Relatively stable with government support	Questionable, as it is dependent on continuous public support	Stable but selective in coverage	Government-backed, but dependent on NICE evaluation and recommendations	Varies widely, dependent on insurance and government programs

*Abbreviations* FDA, Food and Drug Administration; HIRA, Health Insurance Review and Assessment Service; HST, highly specialised technologies; IMF, Innovative Medicines Fund; MFDS, Ministry of Food and Drug Safety; MHRA, Medicines and Healthcare Products Regulatory Agency; NHIS, National Health Insurance Service; NHS, National Health Service; NICE, National Institute for Health and Care Excellence; HST, Highly-Specialised Technologies; LSDP, Life Saving Drugs Program; PBS, Pharmaceutical Benefits Scheme; RDF, Rare Disease Fund; TGA, Therapeutic Goods Administration

### Australia

A national single-payer funding system, *Medicare* serves as the publicly funded universal health insurance scheme in Australia, supplemented by the Pharmaceutical Benefits Scheme (PBS), which aids in covering expenses for certain medications and treatments [41]. The Pharmaceutical Benefits Advisory Committee (PBAC), an independent expert body appointed by the government, employs specific criteria, including cost-effectiveness, to assess a medicine’s eligibility for inclusion in the PBS [41]. While the consideration of cost-effectiveness is pivotal for managing budgets, it poses a challenge in evaluating drugs for rare diseases due to their limited evidence base on effectiveness and higher pricing, influenced by extensive research costs and reduced competition in smaller markets [42]. Accordingly, Australia established the LSDP in 1995 as a complementary initiative to the PBS. The LSDP aims to broaden access to high-cost drugs intended for treating rare diseases, acknowledging the unique challenges posed by such medications within the healthcare landscape. As of 2023, 17 medicines are subsidised via the LSDP [13].

Risk-sharing agreements are commonly used by the PBS and LSDP to manage certain risks and uncertainties with new orphan drugs. Sponsors may voluntarily propose risk-sharing agreements with are captured through a legal deed of agreement that is negotiated between the sponsor and the Government. Some financial risk share agreements can be class deeds where sponsors share the risk based on market share. While such mutual agreements remain confidential, the majority of agreements are likely to be financial-based agreements which include price-volume, rebate or discount-based schemes [43]. However, a hybrid of financial and outcome-based agreements is also possible [44].

Specifically, for the LSDP however, the usage of outcome-based risk-sharing agreements are referenced [45]. These agreements allow funding under the condition that ongoing data collection assesses the drug’s impact on the disease. Price adjustments might occur if emerging data suggests the drug’s efficacy differs from initial assumptions. In the past, LSDP mirrored PBS by implementing a policy to progressively reduce medicine prices on specific listing anniversaries [46]. However, as of June 2022, this policy within LSDP has been discontinued [47]. For orphan drugs under the LSDP, periodic reviews 24 months post-listing remain a crucial aspect of assessing medication usage, clinical benefits, and financial impacts [45]. Recommendations post-review may involve modifying eligibility criteria, adjusting risk-sharing arrangements, altering data collection scopes, referring the medication to PBAC for PBS listing consideration, or even removing it from the LSDP listing [45].

In 2022, Zolgensma® was approved for SMA by listing under the PBS, saving approximately 20 patients AUS$ 2.5 million [48]. In the following year, the scheme was expanded to include pre-symptomatic babies as well, thus extending the subsidy to an additional 15 babies [49]. A cost-minimisation approach was taken, where PBS received a substantial unlisted discount from the sponsor [50]. An outcome-based risk sharing agreement was also established, which encompassed an unspecified rebate on the cost over at least 5 years, following circumstances of a patient’s death and the failure to meet certain developmental milestones [51].

### Singapore

In January 2018, the Singapore government began mulling over the possibility of establishing a separate fund to better support children with rare diseases and their families [52]. Policy discussions culminated in the creation of the RDF, launched in July 2019, to fund five medicines used for the treatment of three rare disease conditions [3]. With an initial endowment of S$70 million, the charity fund operates by combining government-matching contributions with community donations: for every S$1 donated by the public, the government contributes S$3 (3-to-1 matching). According to the then Senior Minister of State for Health, Mr Edwin Tong, the donation matching approach was adopted to galvanise the larger community to “jointly support these patients and their families as part of our caring and inclusive society” [53]. The policy’s focus on collective action and shared responsibility mirrors the core principles of the “Many Helping Hands” approach, a community-based framework that encourages collaboration among stakeholders to address social welfare issues [54].

Although the RDF’s initial focus was directed towards five specific treatments, its non-restrictive framework allows for future expansion to include a wider range of conditions and therapies. In November 2019, the RDF was expanded to cover Pompe disease, a rare inherited neuromuscular disorder where patients can incur medical expenses exceeding S$500,000 each year [55]. Two years later, the RDF was extended to support the treatment of Mucopolysaccharidosis Type VI as well [56]. As of 2023, the RDF covers five conditions and seven medications [3], and has helped relieve the medical financial burden of nine Singaporean patients [57]. It is important to note that the scope of the RDF caters to a relatively small percentage of families requiring additional financial assistance for high-cost treatments. Other avenues, such as *Medisave*, *MediShield Life*, and *MediFund* [58], exist to assist the majority of individuals with rare diseases by covering treatments and medical bills. However, the extent of financial support available through these avenues for such individuals remains limited and subjected to annual caps.

As of the conclusion of the fiscal year 2022, the RDF had a reported total of S$143 million [59]. There have been numerous calls to expand the scope of the RDF to cover more illnesses. In 2021, Member of Parliament Cheryl Chan called for the RDF to be extended to cover the treatment of Neuroblastoma and Krabbe disease, which are among the 10 most common rare diseases afflicting young children in the world [60]. She further added that patients with rare diseases outside the list of approved conditions and medication, unfortunately, face nothing but “the strictest of processes and a flat rejection”. While there remains a strong desire and momentum to support life-saving treatments for various rare diseases, the truth is that the healthcare financing system “is not designed to support such high-cost treatments” [60]. The government’s position, as explained in a Parliamentary reply, is that increasing donations, particularly from high-net-worth individuals, foundations, and corporate sponsors, remains the key approach to securing additional funds for patients and their families [61].

### South Korea

In South Korea, healthcare revolves around the NHIS, a public insurance program managed by the Ministry of Health and Welfare [62]. South Koreans with adequate income contribute to insure themselves and their dependents in this single-payer system. Introduced in 2000, the Mandatory Designation System necessitates all hospitals and clinics to be designated medical care institutions, obligated to provide services to participants in the NHIS, encompassing nearly the entire population [63]. South Korea made a pivotal shift in its National Health Insurance (NHI) drug reimbursement system in 2007, transitioning from a negative to a positive listing approach [14]. Post 2007, only drugs with confirmed cost-effectiveness became eligible for reimbursement. As a result of this change, obtaining reimbursement for orphan drugs where statistically verifying clinical outcomes is challenging, became more arduous. Between 2007 and 2020, South Korea saw the launch and approval of 168 orphan drugs, with 94 of them making it onto the reimbursement formulary [64].

When considering reimbursement pathways for orphan drugs without alternatives, three potential pathways exist. The pharmacoeconomic evaluation exemption pathway was introduced since May 2015 to improve patient accessibility for anticancer and orphan drugs [29]. For orphan drugs classified as essential drugs (ED) or falling under pharmacoeconomic waiver (PEW) categories, submission of a pharmacoeconomic study is not necessary. Instead, these drugs can be listed by referencing the listed prices of the same drug in the A7 countries (which includes the US, the UK, Italy, Germany, Japan, Switzerland, and France). For ED drugs, the average adjusted price in the A7 country sets the reimbursable price, while for PEW drugs, it is the lowest price among the adjusted A7 country prices [14]. ED classification hinges on meeting four criteria: alternative availability, disease severity, patient count, and clinical efficacy. As for PEW drugs, they must simultaneously demonstrate clinical necessity, and evidence challenges, and be listed in over three A7 countries to qualify. The risk-sharing agreement (RSA) pathway is specifically designated for anticancer drugs and orphan drugs lacking alternatives or therapeutically equivalent options [14]. However, within this subset, only those drugs addressing life-threatening critical diseases are eligible to pursue the RSA route. In each pathway, the price for reimbursement gets decided by a committee at the Health Insurance Review and Assessment Service (HIRA), where price negotiation with the NHIS to agree on its budget impact ensues [65]. At present, no special fund for rare disease medications exists in South Korea, although some conditions and drugs are covered under the NHIS [66].

Specific to Zolgensma®, authorities in South Korea studied the results of the available clinical trials and found convincing long-term therapeutic effect that was maintained more than seven years after once dose administration of Zolgensma® [67]. As such, since August 2022, the drug Zolgensma® is covered under the NHIS and patients who require it only have to pay 5.98 million won (around US$4400) despite the drug’s marketed price of 2 billion won (around US$1.5 million) [68]. Patients who receive the drug must consent to a five-year follow-up for regular evaluations of response as part of the government’s effort to continually re-evaluate the usefulness and cost-effectiveness of insured drugs.

### United Kingdom

The National Health Service (NHS) stands as the UK’s publicly funded healthcare system, operating on the core principles of universality and free access to care for all, regardless of nationality or immigration status [69]. As a single-payer system, it covers primary, emergency, and compulsory healthcare at no cost to individuals. Within the NHS framework, the NICE evaluates health technologies based on evidence-based assessments of their effectiveness, safety, and cost-effectiveness. NICE’s role is to ascertain if proposed healthcare expenditures within the NHS offer superior value compared to alternative treatments. Their evaluation involves analysing the cost and benefit of new treatments relative to existing ones, often considering interventions costing less than £20,000 per Quality-Adjusted Life Year (QALY) as cost-effective, allowing some flexibility up to £30,000 per QALY [70]. Notably, once NICE approves a treatment, the NHS is mandated to provide funding for it.

Specific to rare diseases, gaining approval for expensive orphan drugs often faces hurdles due to insufficient evidence for smooth endorsement by the NICE. In 2021, England’s Rare Disease Framework aimed to address this inequality by refining the technology approval process [71]. The changes within the Highly Specialised Technologies (HST) Programme give more weight to health benefits in severe conditions, offer flexibility when evidence generation is challenging, and offer a higher cost-effectiveness threshold of £300,000 per QALY [72]. However, typically, “no more than 300 people in England are eligible for the technology in its licensed indication and no more than 500 across all its indications”, and there should be no other drug options for patients [73]. NICE also considers ‘severity modifiers’ in its appraisals, whereby if the absolute QALY shortfall or proportional QALY shortfall scores are high enough, a QALY weight is applied, effectively increasing the cost-effectiveness threshold [74].

Additionally, the Innovative Medicines Fund (IMF), modelled on the reformed Cancer Drugs Fund (CDF), supports early access to promising treatments for any condition, including rare diseases [15]. With a £340 million annual grant, the IMF provides interim funding for drugs with uncertain clinical and cost-effectiveness. Data collection via trials and studies aims to fill evidence gaps. Negotiations on pricing occur within a value-based framework to strive for cost-effectiveness [75]. However, drugs not deemed superior or cost-effective compared to existing treatments during this evaluation may not receive additional funding. Manufacturers would then bear the financial responsibility for patient access if NICE does not recommend the drug [76]. The approach, though the timeline of patient funding is uncertain, seeks to incentivise high-risk, potentially breakthrough treatments by attracting innovative manufacturers to invest in substantial therapeutic advancements.

Zolgensma®’s successful listing as a subsidised drug under the NHS in 2021 served as the inspiration for the creation of the IMF [77]. A confidential commercial discount was agreed upon, which potentially lowered the Incremental Cost Effectiveness Ratio (ICER), allowing Zolgensma® to be approved under the HST Programme. An outcome-based risk-sharing agreement was set up, linking payment for the drug to substantial clinical advancements. The payment spans five years, and if the therapy falls short of delivering expected clinical outcomes, a partial refund will be issued [78].

### United States

The US’s approach involves a combination of federal and state programs, private insurance, pharmaceutical company initiatives, and non-profit organizations. A signature initiative is the Orphan Drug Act of 1983, which was signed into law and allows the FDA to grant certain drugs or biological products an orphan drug designation [16]. This provides incentives such as tax credits for clinical research, grant funding, assistance in clinical research design, and seven years of market exclusivity upon drug approval for drugs used to treat rare (or orphan because they have been typically neglected) diseases [32], although some have criticized this to be overly lucrative for drug manufacturers [79]. The US National Institutes of Health (NIH), particularly through the National Center for Advancing Translational Sciences (NCATS) and its Office of Rare Diseases Research (ORDR), also plays a significant role in funding and conducting research on rare diseases. The Rare Diseases Clinical Research Network is an initiative that involves collaboration between the NIH, patient advocacy groups, and clinical researchers. Suffice to say, all these efforts ensure constant innovation and a steady pipeline of drug development to change the disease course of rare disease sufferers.

In the US, private health insurance is a major contributor to covering the costs of treatments, including those for rare diseases [80]. However, coverage and out-of-pocket costs can vary significantly and the health systems can be challenging to navigate. An analysis of out-of-pocket spending on orphan drugs from 2013 to 2018 also found an increasing trend (almost doubling from 2013 to 2018) and a higher burden on payers and families despite private insurance coverage [80]. Parents and guardians of children with SMA have also reflected drawn out processing coverage decisions by insurance companies, a lack of transparency in the claims and preauthorization processes and being dependent on employment insurance for coverage [81].

In terms of government-funded health insurance programs (*Medicaid* and *Medicare*), they provide coverage for certain individuals, including those with disabilities and the elderly. They may cover some treatments for rare diseases, depending on the state and specific policy details. In particular, the Affordable Care Act (ACA), also known as Obamacare, has provisions that impact rare disease patients, such as prohibiting insurance companies from denying coverage due to pre-existing conditions, which includes many rare diseases [82]. However, entry criteria for *Medicaid* relies on family income and assets that varies from one state to another [83].

Also worth mention is the numerous non-profit organizations in the US that provide support for rare disease research and advocate for patients [84]. These organizations often fundraise to support research, increase awareness, and assist patients with accessing and affording treatments.

## Discussion

Comparing these high-performing countries’ approaches to rare disease funding reveals a fine balancing act between creating patient access and weighing budgetary impacts. While some countries have well-documented policies and reimbursement mechanisms for rare diseases, others lack detailed studies on the effectiveness and sustainability of their funding models. Identifying these gaps would help to direct future research efforts towards areas that require additional investigation. Singapore’s approach resembles South Korea’s practice of exempting certain orphan drugs from cost-effectiveness analysis. There are shared challenges in conducting such analyses for high-cost, rare disease drugs given the infrequency of rare diseases. However, Singapore’s system, unlike South Korea’s, appears slow in adding drugs to its whitelist and lacks a transparent benchmark for selecting orphan drugs within its RDF. Internationally, countries like Australia, the UK, and South Korea also utilize distinct risk-sharing agreements, which Singapore’s framework does not currently emulate. Additionally, the RDF’s reliance on public goodwill and donations admittedly creates funding instability, and the absence of an early access mechanism or a real-world data monitoring system for orphan drugs prolongs approval processes, contrasting with practices elsewhere. This would inadvertently result in inequities, particularly for patients with non-listed rare diseases. Similarly, in the US, the fragmented healthcare system results in varied access and high out-of-pocket costs, despite robust research funding and the Orphan Drug Act providing incentives for drug development.

To address the high costs (and current uncertain long-term efficacy) of treatments for rare diseases like SMA, countries can benefit from implementing risk-sharing agreements with pharmaceutical companies. An important driver for uncertainty is the sustainability of remission, as it could range from 1 year to life-long effects. A risk-sharing agreement approach involves the government or healthcare providers negotiating with drug manufacturers to agree on terms that link the payment for the drugs to their performance in the real world or to specific outcomes. Given the paucity of long-term effectiveness data for treatments for rare diseases, making future payments conditional on the actual health outcomes and cost savings achieved would be a financially prudent approach for governments. Moreover, a recent study conducted found that pharmaceutical manufacturers and public payers had high interest in outcomes-based agreements and understood their role in facilitating timely market access for patients in need, provided that they are carefully designed to ensure value [85].

The core advantage of this model lies in its potential to make expensive therapies more accessible while managing financial risks. These agreements can be structured in various ways, such as paying for a drug only if it meets certain efficacy benchmarks or spreading the cost over time based on continued patient benefit. This strategy aligns the interests of public healthcare systems, patients, and pharmaceutical companies, ensuring that payment is contingent on the actual value provided by the treatment. Moreover, a payment-by-instalment method, which has been contemplated elsewhere, helps to spread the cost of these high-priced therapies over a period of time, thereby easing the immediate impact on healthcare budgets [86].

Such arrangements are not new, and outcome-based rates, tied to short- and long-term outcomes of patients post-treatment, have been successfully established for other high-cost gene therapies in the US and elsewhere [87]. In fact, risk-sharing agreements to mitigate investment risk for high-cost drugs are growing at an annual rate of 24% since 2012 [88]. Such agreements also benefit from the involvement of various stakeholders such as patients, healthcare providers, payers, policymakers, and manufacturers. In the case of Zolgensma®, a risk-sharing agreement could involve initial partial payment, with subsequent payments contingent upon the drug demonstrating a certain level of effectiveness in patients. Similar to the South Korean approach [28], such agreements also encourage pharmaceutical companies to invest in long-term studies and data collection to validate the effectiveness of their products.

Nonetheless, outcome-based pricing shifts some financial risks to drug manufacturers, who may only receive full payment upon proven effectiveness of the treatment. This risk might lead to higher initial pricing or reluctance from manufacturers to engage in further research and development of therapeutics for other rare diseases. Likewise, outcome-based pricing models require certain alterations in traditional healthcare insurance practices, which may be resistant to change due to established protocols and risk aversion. Defining reasonable outcomes that accurately reflect the effectiveness of the treatment can also be a challenge, especially when it comes to gene therapies where studies are still ongoing and long-term effects are not yet fully understood. The added administrative burden for healthcare providers and insurers to keenly track and monitor patient outcomes could also potentially impede the overall efficiency of the healthcare system [89].

Despite these challenges, the potential benefits of risk-sharing agreements in managing the financial burden of expensive rare disease treatments make them a compelling option for most high-performing health systems. They offer a pragmatic approach to balancing cost, access and innovation in healthcare. For the future, countries should also share best practices and data across borders as this can enhance global understanding of rare disease management and funding. Collaborative efforts can better drive innovation and harmonize approval and reimbursement processes, benefiting patients worldwide.

### Limitations

Despite performing a comprehensive literature search across multiple databases and grey literature sources, there are some shortcomings to the present scoping review and policy analysis. First, in spite of best attempts at ensuring that the search strategy and literature consulted were wide-ranging, certain policy documents and commercial agreements may be confidential and not privy to the public. As such, the scoping review may not cover all relevant policy measures, considerations and outcomes, which could result in an incomplete picture of the strategies and their effectiveness in managing access and the cost of rare disease treatments. Second, the specific focus on high-income countries with advanced healthcare systems limits the generalizability of our findings to LMICs. In particular, LMICs face various challenges and have different healthcare infrastructure and funding mechanisms, which are not addressed in our review. Third, the overt lack of standardized outcome measures (e.g. cost-effectiveness ratios) across the reviewed studies impeded close comparisons of the relative effectiveness and impact of different funding strategies. Future research should prioritize the development and use of uniform metrics to enhance the comparability and synthesis of findings.

## Conclusion

Through this scoping review and policy analysis, we recognize that while no country has effectively addressed the challenge of financing rare diseases, the majority have clearly acknowledged that fairness of access is a moral obligation of public health systems. Developed countries and high-performing health systems should further explore and implement outcome-based risk-sharing agreements to balance immediate costs with long-term benefits for patients afflicted by rare diseases. These agreements can ensure that payments are contingent on real-world efficacy, spreading financial risk and encouraging ongoing data collection. Given the rarity and substantial expense of treatments for rare diseases, the most feasible solution seems to lie in improving national healthcare insurance schemes. Equitable rare disease funding should be an area of continued interest and research.

### Electronic supplementary material

Below is the link to the electronic supplementary material.

Supplementary Material 1

## Data Availability

The authors confirm that the data supporting the findings of this study are available within the article and its supplementary material.
