# Supplementary material for: Comparative policy analysis of national rare disease funding policies in Australia, Singapore, South Korea, the United Kingdom and the United States: a scoping review
Source: Health Econ Rev. 2024 Jun 19;14:42. doi: 10.1186/s13561-024-00519-1 (PMC11186122; doi:10.1186/s13561-024-00519-1)
Supplement: Supplementary file 1 — Supplementary Material 1 [file 13561_2024_519_MOESM1_ESM.docx]

**Supplementary Material**

Table S1. Full search strategy for the electronic databases

*Embase Search Strategy*

| **No.** | **Search Terms** |
| --- | --- |
| #1 | 'orphan disease*':ti,ab OR 'orphan*':ti,ab OR 'rare condition*':ti,ab OR 'rare disorder*':ti,ab OR 'rare disability*':ti,ab OR 'rare*':ti,ab OR 'neglected disease*':ti,ab OR 'undiagnosed disease*':ti,ab OR 'low frequency disease*':ti,ab OR 'life-threatening disease*':ti,ab OR 'debilitating disease*':ti,ab OR 'severe disease*':ti,ab OR 'intractable disease*':ti,ab OR 'rare disease*':ti,ab |
| #2 | 'orphan disease'/exp |
| #3 | 'rare disease'/exp |
| #4 | #1 OR #2 OR #3 |
| #5 | 'public*':ti,ab OR 'health*':ti,ab OR 'community*':ti,ab OR 'national*':ti,ab OR 'government*':ti,ab |
| #6 | 'public health'/exp |
| #7 | 'community health'/exp |
| #8 | 'government'/exp |
| #9 | #5 OR #6 OR #7 OR #8 |
| #10 | 'policy*':ti,ab OR 'policies*':ti,ab |
| #11 | 'policy'/exp |
| #12 | #10 OR #11 |
| #13 | 'fund*':ti,ab OR reimbursement* OR 'insurance*':ti,ab OR 'financ*':ti,ab OR 'contrib*':ti,ab OR 'donat*':ti,ab OR 'grant*':ti,ab OR 'sponsor*':ti,ab OR 'support':ti,ab |
| #14 | 'fund' |
| #15 | 'funding'/exp |
| #16 | 'insurance'/exp |
| #17 | #13 OR #14 OR #15 OR #16 |
| #18 | 'orphan medicinal product*':ti,ab OR 'orphan product*':ti,ab OR 'orphan subset*':ti,ab OR 'orphan indication*':ti,ab OR 'highly specialized technolog*':ti,ab OR 'priority review drug*':ti,ab OR 'orphan drug*':ti,ab OR 'orphan drug production*':ti,ab |
| #19 | 'orphan drug'/exp |
| #20 | #18 OR #19 |
| #21 | #4 OR #20 |
| #22 | #9 AND #12 AND #17 AND #21 |

*Medline Search Strategy*

| **No.** | **Search Terms** |
| --- | --- |
| #1 | (Orphan disease* or Rare condition* or Rare disorder* or Rare disability* or Neglected disease* or Undiagnosed disease* or Low-frequency disease* or life-threatening disease* or debilitating disease* or severe disease* or intractable disease* or Rare Disease*).mp. |
| #2 | (Orphan disease* or Rare condition* or Rare disorder* or Rare disability* or Neglected disease* or Undiagnosed disease* or Low-frequency disease* or life-threatening disease* or debilitating disease* or severe disease* or intractable disease* or Rare Disease*).ti,ab. |
| #3 | exp rare disease/ |
| #4 | exp orphan disease/ |
| #5 | 1 or 2 or 3 or 4 |
| #6 | (public* or health* or community* or National* or Government*).mp. |
| #7 | (public* or health* or community* or National* or Government*).ti,ab. |
| #8 | exp public health/ |
| #9 | exp community health/ |
| #10 | exp government/ |
| #11 | 5 or 6 or 8 or 9 or 10 |
| #12 | (policy* or policies*).mp. |
| #13 | (policy* or policies*).ti,ab. |
| #14 | exp policy/ |
| #15 | 12 or 13 or 14 |
| #16 | (fund* or reimbursement* or insurance* or financ* or contrib* or donat* or grant* or sponsor* or support*).mp. |
| #17 | (fund* or reimbursement* or insurance* or financ* or contrib* or donat* or grant* or sponsor* or support*).ti,ab. |
| #18 | exp fund/ |
| #19 | exp funding/ |
| #20 | exp insurance/ |
| #21 | 16 or 17 or 18 or 19 or 20 |
| #22 | (Orphan medicinal product* or Orphan product* or Orphan subset* or Orphan indication* or Highly specialized technolog* or Priority review drug* or Orphan Drug* or Orphan Drug Production*).mp. |
| #23 | (Orphan medicinal product* or Orphan product* or Orphan subset* or Orphan indication* or Highly specialized technolog* or Priority review drug* or Orphan Drug* or Orphan Drug Production*).ti,ab. |
| #24 | exp orphan drug/ |
| #25 | 22 or 23 or 24 |
| #26 | 5 or 25 |
| #27 | 11 and 15 and 21 and 26 |

*Cochrane Search Strategy*

| **No.** | **Search Terms** |
| --- | --- |
| #1 | 'orphan disease*':ti,ab OR 'orphan*':ti,ab OR 'rare condition*':ti,ab OR 'rare disorder*':ti,ab OR 'rare disability*':ti,ab OR 'rare*':ti,ab OR 'neglected disease*':ti,ab OR 'undiagnosed disease*':ti,ab OR 'low frequency disease*':ti,ab OR 'life threatening disease*':ti,ab OR 'debilitating disease*':ti,ab OR 'severe disease*':ti,ab OR 'intractable disease*':ti,ab OR 'rare disease*':ti,ab |
| #2 | mh 'orphan disease' |
| #3 | mh 'rare disease' |
| #4 | #1 or #2 or #3 |
| #5 | 'public*':ti,ab OR 'health*':ti,ab OR 'community*':ti,ab OR 'national*':ti,ab OR 'government*':ti,ab |
| #6 | mh 'public health' |
| #7 | mh 'community health' |
| #8 | mh 'government' |
| #9 | #5 OR #6 OR #7 OR #8 |
| #10 | 'policy*':ti,ab OR 'policies*':ti,ab |
| #11 | mh policy |
| #12 | #10 OR #11 |
| #13 | 'fund*':ti,ab OR reimbursement* OR 'insurance*':ti,ab OR 'financ*':ti,ab OR 'contrib*':ti,ab OR 'donat*':ti,ab OR 'grant*':ti,ab OR 'sponsor*':ti,ab OR 'support':ti,ab |
| #14 | mh 'fund' |
| #15 | mh 'funding' |
| #16 | mh 'insurance' |
| #17 | #13 OR #14 OR #15 OR #16 |
| #18 | 'orphan medicinal product*':ti,ab OR 'orphan product*':ti,ab OR 'orphan subset*':ti,ab OR 'orphan indication*':ti,ab OR 'highly specialized technolog*':ti,ab OR 'priority review drug*':ti,ab OR 'orphan drug*':ti,ab OR 'orphan drug production*':ti,ab |
| #19 | mh 'orphan drug' |
| #20 | #18 OR #19 |
| #21 | #4 OR #20 |
| #22 | #9 AND #12 AND #17 AND #21 |
